# Quantifying an Upper Extremity Everyday Task With 3D Kinematic Analysis in People With Spinal Cord Injury and Non-disabled Controls

**DOI:** 10.3389/fneur.2021.755790

**Published:** 2021-10-15

**Authors:** Lamprini Lili, Katharina S. Sunnerhagen, Tiina Rekand, Margit Alt Murphy

**Affiliations:** ^1^Institute of Neuroscience and Physiology, Clinical Neuroscience, Rehabilitation Medicine, University of Gothenburg, Sahlgrenska Academy, Gothenburg, Sweden; ^2^Department of Neurocare, Sahlgrenska University Hospital, Gothenburg, Sweden; ^3^Department of Neurology, Haukeland University Hospital, Bergen, Norway; ^4^Department of Occupational Therapy and Physiotherapy, Sahlgrenska University Hospital, Gothenburg, Sweden

**Keywords:** spinal cord injury, movement analysis, upper extremity (arm), drinking task, quality of movement, assessment, kinematics, functioning

## Abstract

**Objectives:** Upper extremity function after spinal cord injury (SCI) is an important factor for performance of activities of daily living. An objective assessment of upper extremity function preferably in purposeful daily tasks is essential in understanding its impact on real-life activities. This study aimed to identify which movement parameters of upper extremity, measured by kinematic analysis during a purposeful daily task, are impaired in people with cervical or thoracic SCI.

**Materials and Methods:** The study included 29 adults (mean 59.5 years, 9 women and 20 men) with cervical (*n* = 19) or thoracic (*n* = 10) established complete (*n* = 15) or incomplete (*n* = 14) SCI, and 54 non-disabled controls with commensurable age and sex (mean 59 years, 15 women, 39 men). The 3D kinematic data were captured with a five-camera system during a standardized unilateral daily task (drinking from a glass). In SCI, the upper extremity functioning of each arm was assessed with Action Research Arm Test (ARAT). Having a full score in ARAT indicated full functioning; a score of <57 points indicated limited functioning. Kinematic data from full functioning arms (*n* = 27) and limited functioning arms (*n* = 30) in SCI were compared with the non-dominant arms (*n* = 54) in controls.

**Results:** In the limited upper extremity functioning group, movement time, smoothness, arm abduction, wrist angle, trunk displacement, and inter-joint coordination, but not peak velocity of the hand, angular velocity of elbow, and relative time to peak velocity, all differed from controls. In the full upper extremity functioning group, arm abduction alone was significantly different from controls.

**Conclusions:** The findings demonstrate that apart from measures of peak velocity, kinematic measures of movement quality including movement time, smoothness, trunk displacement, and joint angles are impaired in people with limited upper extremity functioning after SCI. The study provides robust results applicable to a representative population of individuals with established cervical or thoracic SCI. The results suggest that kinematic analysis might be useful for those with limited functioning in order to get a better understanding of the specific movement impairments in daily tasks after SCI.

## Introduction

Spinal cord injury (SCI) is a life-changing condition resulting in a partial or complete loss of sensory and/or motor function below the level of injury. In about half, the SCI will impact upper extremity functioning in activities of daily living (1, 2). The upper extremity impairment involves both arms and asymmetries are common, particularly in incomplete cervical SCI (3). The prevalence of incomplete SCI has been increasing during the last decades and is estimated to be approximately 60% (1, 2, 4). These trends in SCI highlight the need to assess movements and functioning in daily activities and tasks considering possible differences in function between the arms.

The International Classification of Functioning, Disability, and Health (ICF) provides a universally accepted framework to describe functioning in people with disabilities (5). The use of a wider ICF perspective in SCI has been advocated for many years, although the traditional clinical assessment often focuses on the anatomical localization of the injury and the severity of the neurological impairment (6–8). Functional activity level assessment is, however, an important aspect of rehabilitation and will provide a better understanding of limitations in daily life activities (9–11). The Action Research Arm Test is one such performance-based standardized clinical assessment scale that can be used to evaluate activity capacity level in SCI (12). The remaining limitation in clinical scales is, however, that scoring relies on the observation of a clinician and pre-set categories of the scale and will therefore be limited in capturing more refined alterations in quality movement (13, 14).

Kinematic analysis, using camera-based high-speed optoelectronic systems, is a recommended tool for precise and detailed 3D analysis of movement quality and performance (14). Even though the kinematics are increasingly used in some areas, such as stroke, the use in SCI has been sparse, and mainly limited to smaller studies with complete SCI at the neurological level of C5-C7 (15–20). While most of the kinematic studies evaluate simple reaching or pointing, the analysis of a purposeful daily task, such as, drinking, has a higher ecological validity (20, 21).

The aim of this study was to determine whether and which upper extremity kinematic measures obtained during the drinking task are impaired in people with different levels of functioning (full or limited) after cervical or thoracic SCI when compared with non-disabled control group.

## Materials and Methods

### Participants

**Figure 1:**
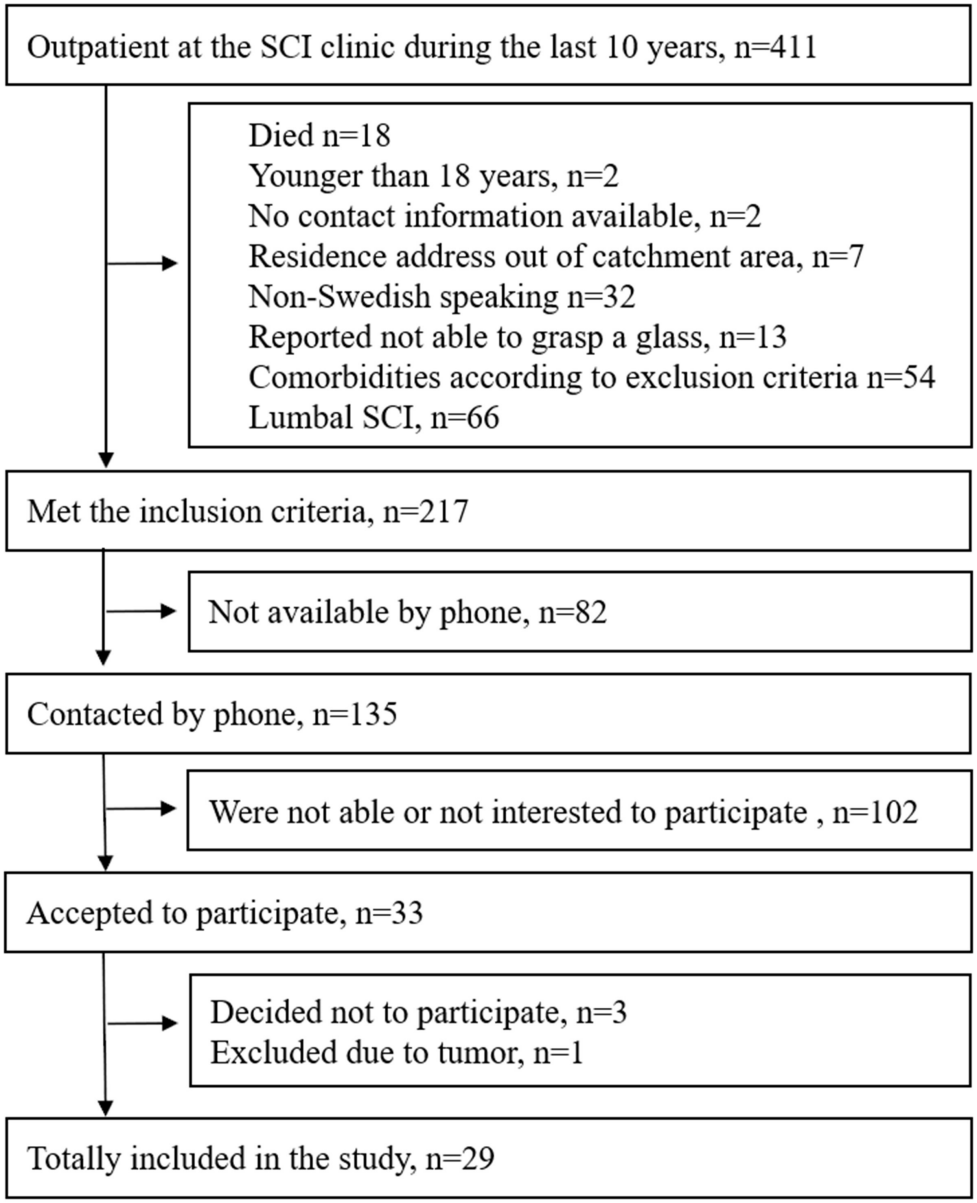
Flow chart of inclusion process.

Individuals with SCI who had been in contact with the outpatient clinic at Sahlgrenska University Hospital during the last 10 years (2006–2016), were screened for potential inclusion. The inclusion criteria were as follows: having had a cervical or thoracic SCI at least 1 year prior to inclusion, older than 18 years, having a residence address within the geographical catchment area, and able to involve the arm(s) in activities of daily living. The exclusion criteria were as follows: other neurological or musculoskeletal conditions that could influence the upper extremity functioning; comorbidities, such as, major depression, psychosis, or other mental disorders; not able to perform the drinking task with any arm; and not being able to communicate in Swedish. The inclusion process is shown in Figure 1.

Consequently, 29 individuals (9 women and 20 men, mean age of 59.5 years, range 33–81) with complete or incomplete (*n* = 15/14), cervical or thoracic (*n* = 19/10) SCI with motor level varying between C5 to T12 (according to International Standards for Neurological Classification of Spinal Cord Injury, ISNCSCI) were included in this cross-sectional study between 2018 and 2019. Eleven participants (15 hands) had had a previous hand surgery. The mean height and weight of the entire cohort of SCI was 176 cm and 76 kg, respectively.

In addition, 54 non-disabled controls with commensurable age (mean 59 years, range 26–81) and sex distribution (15 women and 39 men) who did not present any upper extremity dysfunction were included as the reference group. The mean height and weight of the controls were 176 cm and 71 kg, respectively.

The study was approved by the Swedish Ethical Review Authority (408-17), and informed written consents were obtained from all participants prior to inclusion. The research was performed in accordance with the Declaration of Helsinki. The study was registered at researchweb.org (https://www.researchweb.org/is/vgr/project/260901) prior to participant enrollment. The reporting of this study conforms to the Strengthening the Reporting of Observational Studies in Epidemiology (STROBE) statement (22).

### Kinematic Movement Analysis

**Figure 2:**
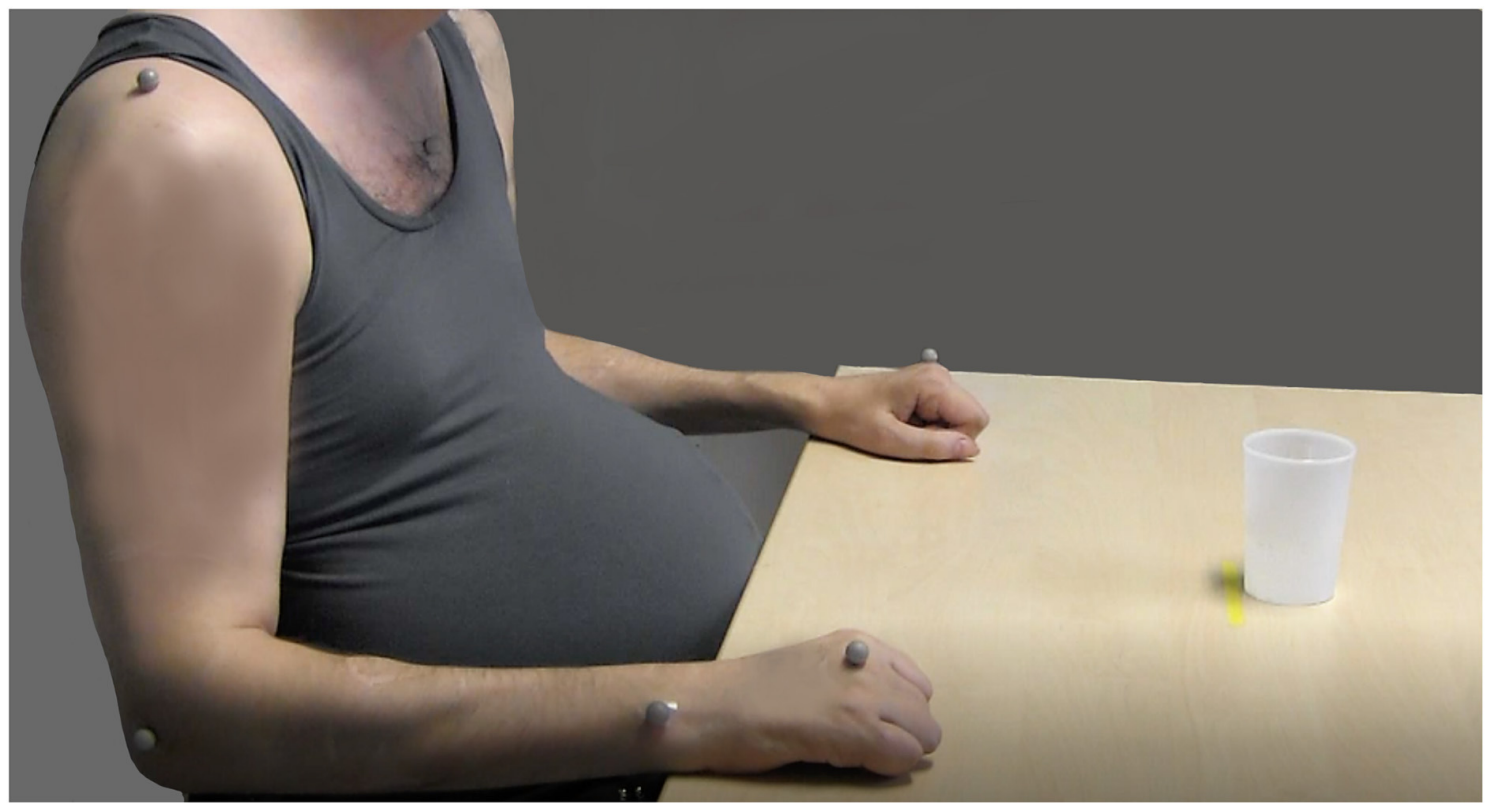
The initial sitting position and marker placements on the body for the kinematic drinking task shown in a participant with spinal cord injury (SCI) (the markers on the head, left shoulder, and the drinking glass are not visible) (source corresponding author).

A five-camera optoelectronic motion capture system (Pro Reflex Motion Capture System, MCU240 Hz, Qualisys AB, Sweden) was used for 3D kinematic analysis. The cameras placed around the measurement area emit infrared light signals at high speed that are reflected by the passive markers placed on defined anatomical landmarks on the body. Eight reflective spherical markers (12 mm) were attached on the skin using double-sided adhesive tape, and one marker was attached on the drinking cup. Markers were placed on both hands (third metacarpophalangeal joint), the tested wrist (the styloid process of ulna), the tested elbow (lateral epicondyle), both shoulders (middle part of acromion), the thorax (upper part of sternum), and the face (notch between eyebrows) (Figure 2) (23, 24). Kinematic 3D data from markers were automatically identified and transferred for offline custom-made analysis using MATLAB (The Math Works Inc.) software (24). Kinematic data were filtered using a 6 Hz second-order Butterworth filter in both forward and reverse directions, resulting in a zero-phase distortion and fourth-order filtering (23, 24).

A standardized drinking task protocol with good test–retest reliability was applied in this study (23–25). In short, the participants were instructed to sit in front of a table with their back against the chair back (Figure 2). The chair and table height were adjusted to attain 90° knee and hip flexion, 90° elbow flexion, while the upper arm was in the vertical and forearm in the horizontal positions. The wrist joint was aligned with the table edge, and the palm of the hand was resting on the table. This standardization of the initial sitting position resulted in that for each participant the drinking cup was approximately at 75% of the arm's length. Participants using the wheelchair remained sitting in their own chair but the testing was standardized as described above, as far as possible, by adjusting the table height. If participants had difficulties maintaining their sitting balance during the task performance, they were allowed to stabilize their body by using the non-tested arm. A hard-plastic glass containing 100 ml of water was placed 30 cm from the table edge in the midline of the body, behind a line marked on the table. If the participant proved unable to use the standard glass, several other types of drinking cups were made available for use (hard-plastic wine glass, plastic cup with a handle).

The drinking task included five phases: (i) reaching phase included lifting the hand from the table and grasping the glass, (ii) forward transport phase included securing the grasp and transporting the glass to the mouth, (iii) drinking phase included taking one sip of water, (iv) backward transport phase included moving the glass back on the table and releasing the grasp, (v) and returning phase, moving the hand back to the initial position on the table edge (23, 24). The participants were asked to perform the drinking task 8–10 times unimanually and starting with their dominant arm at a comfortable, self-paced speed. For the statistical analysis a mean of all trials was calculated for each kinematic variable separately for each arm (25).

### Kinematic Variables

The kinematic variables obtained from the drinking task included measures, such as, movement times, movement smoothness, movement velocity, and strategy measures, as well as measures characterizing movement patterns including joint angles.

Movement time was calculated for the entire drinking task (total movement time) as well as for each movement phase separately from the hand marker. The start and end of movement was defined by the velocity of the hand marker (2% of the maximum velocity) (24). Exact definitions of the movement phases were described in detail in previous publications (23, 24).

Movement smoothness was defined as the number of movement units (NMU) identified from the tangential velocity profile of the hand marker. NMU was calculated separately for the first two (reach and forward transport) and the last two movement phases (back transport and return), and summed as total NMU. A movement unit was defined as the difference between a local minimum and the next maximum velocity value that exceeded the amplitude limit of 20 mm/s, where the time between two subsequent peaks had to be at least 150 ms (23). These peaks indicate repeated accelerations and decelerations during movement performance and reflect efficiency and smoothness of movement (26). Peak hand velocity and percentage of time to peak velocity (relative acceleration time) were calculated for the reaching phase using the hand marker. The peak elbow angular velocity during elbow extension in reaching phase was computed from the angular data described below.

Movement pattern measures included maximal elbow extension in reaching, maximal wrist angle in reaching, and forward transport phase, and maximal arm abduction in drinking phase. The joint angles for the wrist and elbow were determined by the angles between the vectors joining the hand, wrist, elbow, and shoulder markers. A smaller elbow angle value indicated a position closer to extension and a larger wrist angle value indicated a position closer to dorsal wrist flexion. The shoulder abduction was defined as the angle between the vectors joining the shoulder and elbow markers and the vertical vector from the shoulder marker toward the hip projected into the frontal plane. Inter-joint coordination was calculated as a cross-correlation between the shoulder flexion and elbow extension joint angles during the reaching phase. Joint motion between shoulder and elbow is tightly coupled during reaching in non-disabled controls (23). Trunk displacement was defined as the maximal forward displacement of the thorax in the sagittal plane from the initial position.

### Level of Upper Extremity Functioning

The Action Research Arm Test (ARAT) was used to assess the upper extremity functioning in individuals with SCI (27, 28). ARAT includes 19 items divided into four subscales (grasp, grip, pinch, and gross movement). Majority of items assess the ability to grasp and move objects of different shapes and sizes into different vertical or horizontal locations in the arm workspace. ARAT has excellent reliability and validity in stroke (29, 30) and has been increasingly used in SCI (12, 31). A maximum score of 57 is received when all items of the scale are performed within a 5-s time limit while using a correct movement and grasp configuration. The ARAT was scored separately for the dominant and non-dominant upper extremity. In this study, the arms that were scored with <57 points were classified as limited upper extremity functioning, while the arms that were scored with a full score of 57 points were classified as full upper extremity functioning.

### Other Background Clinical Characteristics

The International Standards for Neurological Classification of Spinal Cord Injury (ISNCSCI) examination was used to determine the neurological level of SCI alongside the American Spinal Injury Association (ASIA) Impairment scale (AIS) which was used to classify the completeness of the injury (6) (Table 1). Type of performed hand reconstructive surgery, SCI-related complications to upper extremity like pain, spasms, contractures, edema, and use of assistive devices were recorded according to the International SCI Upper Extremity Data Set (32) (Supplementary Table 1). Independence in self-care was assessed with the Spinal Cord Independence Measure (SCIM III) (33). Body Mass Index (BMI) was calculated from the self-reported body weight and height.

**Table 1:** Characteristics of the individuals with spinal cord injury (SCI) included in the limited and full upper extremity functioning groups.

**Characteristics**	**Limited UE functioning**		**Full UE functioning**	
	**Mean, median or *n***	**SD, IQR or %**	**Mean, median or *n***	**SD, IQR or %**
Age, years, mean	59.3	11.7	59.5	15.5
Sex, *n*				
Female	9	30%	9	33%
Male	21	70%	18	67%
Height, cm, mean	175.0	8.6	177.2	9.7
Weight, kg, mean	72.0	14.4	80.4	14.9
BMI, mean	23.8	4.6	25.5	4.1
Overweight, *n*	9	33%	10	37%
Years since SCI, mean	22.8	16.2	9.9	10.8
Injury type				
Traumatic, *n*	27	90%	16	59%
Non-traumatic, *n*	3	10%	11	41%
Level of SCI				
Cervical, *n*	25	83%	12	44%
Thoracic, *n*	5	17%	15	56%
Completeness of SCI				
AIS A, B, *n*	20	67%	5	18.5%
AIS C, D, E, *n*	10	33%	22	81.5%
Hand surgery, *n*	12	40%	3	11%
SCIM III self-care (0–20 points), median	13	9–18	19	18–20
ARAT (0–57 points), median	43	30–54	57	57–57
Tested upper extremity, n^*^	30		27	
Dominant UE, *n*	14	47%	15	56%
Non-dominant UE, *n*	16	53%	12	44%

* *One person in the limited functioning group was able to perform the drinking task with the dominant arm only. In nine participants the UE functioning was categorized as limited in one arm and full in the other arm according to the Action Research Arm Test*.

*UE, upper extremity; BMI, body mass index; SCI, spinal cord injury; AIS, ASIA Impairment Scale; A, complete injury; B, sensory incomplete, but motor complete injury; C and D, incomplete injury; E, normal motor and sensory function; SCIM III, Spinal Cord Independence Measure; ARAT, Action Research Arm Test, SD, standard deviation; IQR, interquartile range*.

### Statistical Analysis

Statistical analysis was performed using the IBM Statistical Package for Social Sciences (SPSS, version 24). Based on previous studies, mean expected difference in movement time (3 s) between groups with statistical power of 0.80 (*p* = 0.05) required a sample size of 20 in each group (23). Descriptive statistics were calculated for demographic and clinical characteristics.

All but one participant with SCI were able to perform the drinking task both with their dominant and non-dominant arm. Thus, kinematic data was available from 57 arms. The limited upper extremity functioning group (ARAT < 57 points) included data from 30 arms and full upper extremity functioning (ARAT = 57 points) included data from 27 arms. In nine participants, the upper extremity functioning was categorized as limited in one arm and full in the other arm. In non-disabled controls, kinematic data of the non-dominant arm was used in the statistical analysis. This more conservative choice was selected to provide a justified comparison with the SCI groups, which included data both from dominant and non-dominant arms. The possible impact of hand dominance on the results was tested by using interaction terms as described below.

Kinematics of the limited and full functioning groups in SCI were compared with the non-disabled controls by using independent *t*-test. In case of unequal variances between the compared groups, according to the Levene's test of equality, the t-statistics and *p*-values for the equal variances not assumed were used. The level of significance (alpha value) was set to *p* < 0.05. For all significant differences, the strength of difference between groups was determined using the eta squared (η^2^) effect size (ES) estimates (34). The eta squared ranges from 0 to 1 and represents the proportion of variance in the kinematic variable that was explained by the group variable. Cohen's guidelines were followed to interpret the effect size where 0.01–0.05 indicates small effect, 0.06–0.13 moderate effect, and ≥0.14 large effect size (34).

To test the impact of sex (male/female), age (age>61), hand dominance (dominant/non-dominant) and overweight (BMI > 25) factor on kinematic variables, the interaction effect between the group (limited, full, control) and the factor were analyzed with the two-way between-groups analysis of variance. In case non-equal variance between the groups (Levene's test of equality <0.05), a more stringent significance level (*p* < 0.01) for determining the interaction effect was used.

## Results

The summarized characteristics of the limited and full upper extremity functioning groups are displayed in Table 1. The Supplementary Table 1 shows the clinical background characteristics separately displayed for each tested arm for all participants. Age, sex, hand dominance, and BMI distributions were similar in both groups with limited and full upper extremity functioning. None of the tested factors (age, sex, hand dominance, and overweight) showed significant interaction effect on kinematic variables. The level (cervical or thoracic) and the completeness (AIS A–E) of the spinal cord injury varied in both groups, and the limited functioning group included a larger proportion of measurements from individuals with traumatic, complete, and cervical injury than the full functioning group. In the entire sample, due to the deficits in grasping ability, two participants with SCI used a hard-plastic wine glass and two used a plastic cup with a handle.

### Movement Time

The total movement time was significantly longer in the limited functioning group compared with controls (mean difference 2.71 s, large ES = 0.14, Table 2). Specifically, movement time was longer in the reaching phase that also included grasp formation (mean difference 0.22 s, moderate ES = 0.09), in the forward transport phase that included securing the grasp and transporting the glass to the mouth (mean difference 1.51 s, moderate ES = 0.10), and in the back transport phase when the glass was moved back and released on the table (mean difference 0.55 s, large ES = 0.14). There were no differences observed between the full functioning SCI group and non-disabled controls.

**Table 2:** Kinematic variables of the drinking task for limited and full upper extremity functioning groups in SCI, and non-disabled controls.

**Kinematic variables**	**SCI**			**SCI**			**Controls non-dominant arm**	
	**Limited UE functioning (30 arms)**			**Full UE functioning (27 arms)**			**(54 arms)**	
	**Mean**	**SD**	***p*-value (ES)**	**Mean**	**SD**	***p*-value (ES)**	**Mean**	**SD**
**Movement time**, ***s***
Reaching	1.23	0.41	0.009 (0.09)	0.93	0.19	0.050	1.01	0.17
Forward transport	2.71	2.70	0.005 (0.10)	1.22	0.29	0.760	1.20	0.27
Drinking	1.58	0.46	0.114	1.30	0.26	0.061	1.44	0.35
Back transport	2.17	0.78	0.001 (0.14)	1.54	0.32	0.370	1.61	0.32
Returning	1.39	0.81	0.057	1.04	0.23	0.282	1.10	0.23
Total movement time	9.07	4.06	0.001 (0.14)	6.02	0.97	0.154	6.36	1.01
**Smoothness, number of movement units**, ***n***
NMU, total	15.2	12.8	<0.001 (0.16)	6.29	1.22	0.168	5.95	0.96
NMU reach and forward transport	7.75	8.55	0.001 (0.13)	2.37	0.39	0.115	2.24	0.36
NMU back transport and return	7.46	4.85	<0.001 (0.17)	3.92	0.93	0.728	3.84	0.96
**Movement velocity and strategy**
Peak hand velocity reach, mm/s	609	181	0.778	663	108	0.059	619	89.7
Time to PHV reach, %	0.41	0.09	0.094	0.44	0.06	0.851	0.44	0.08
Peak elbow angle velocity reach °/s	91.7	43.0	0.148	105	22.0	0.931	104	21.3
**Movement pattern**
Elbow extension reach, degree	58.0	13.7	0.293	56.7	9.9	0.414	55.1	7.7
Arm abduction drink, degree	48.8	28.9	0.002 (0.12)	35.9	9.3	0.031 (0.06)	30.9	9.8
Wrist angle reach and forward transport, degree	40.3	17.1	<0.001 (0.16)	27.2	6.1	0.701	27.8	5.2
Inter-joint coordination reach, *r*	0.79	0.40	0.038 (0.06)	0.94	0.14	0.612	0.95	0.10
Trunk displacement, cm	6.41	4.22	<0.001 (0.17)	2.39	1.54	0.055	3.15	1.69

*p-value indicates comparison with non-disabled controls; ES statistics are calculated as eta squared (η^2^) for significant differences and are interpreted as 0.01–0.05 small effect, 0.06–0.13 moderate effect, ≥0.14 large effect. UE, upper extremity; NMU, number of movement units; PHV, peak hand velocity; r, Pearson's correlation coefficient; ES, effect size*.

### Movement Smoothness

**Figure 3:**
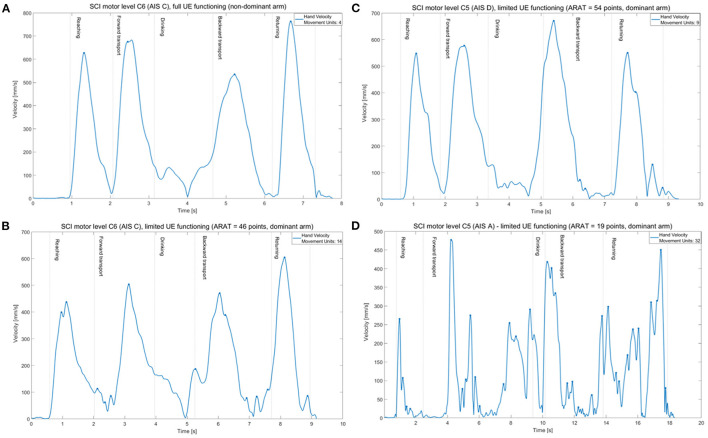
Tangential velocity profiles (hand marker) and number of movement units (marked as dots) of the drinking task shown in one individual from full functioning group **(A)** and three individuals from limited functioning group with different levels of functioning after SCI **(B–D)**.

The limited functioning group showed larger number of movement units indicating less smooth movements compared with non-disabled controls (mean difference 9.27 units, large ES = 0.16, Table 2). The number of movement units was significantly larger both in the first two and last two movement phases. No significant differences were noted between the full functioning SCI group and non-disabled controls. The velocity profiles with indicated number of movement units are illustrated for four representative participants with different levels of functioning after SCI in Figure 3.

### Movement Velocities and Strategy

There were no differences observed between the limited or full functioning group and non-disabled controls for the measures of peak hand velocity, relative time to peak hand velocity, and peak angular velocity of the elbow extension during reaching (Table 2).

### Movement Pattern

The limited functioning group showed significantly larger angle in wrist joint (dorsal flexion) in reaching and forward transport phase (mean difference 12.5°, large ES = 0.16) and larger arm abduction angle in drinking phase (mean difference 17.9°, large ES = 0.12), but no difference was detected for the elbow extension in reaching compared with controls (Table 2). Likewise, a significantly larger trunk displacement (mean difference 3.3 cm, large ES = 0.17) and a weaker inter-joint coordination (Pearson's correlation) between the elbow extension and shoulder flexion in reaching (mean difference 0.16, moderate ES = 0.06) was detected in the group with limited functioning compared with controls. In the full functioning group, the arm abduction angle (mean difference 5.0°, moderate ES = 0.06) was larger compared to controls.

## Discussion

This study demonstrated that kinematic measures of movement time, movement smoothness, and movement pattern including arm and wrist joint angles, inter-joint coordination, and trunk displacement were all impaired in individuals with limited upper extremity functioning after SCI when compared with non-disabled controls. Interestingly, the velocity-related kinematics of tangential and angular peak velocity as well as relative time to peak velocity in reaching did not show significant differences from controls. This means that most, but not all, kinematics are altered after SCI. In contrast, full upper extremity functioning after SCI according to ARAT, i.e., having ability to perform both gross and fine motor tasks timely with a correct grasp configuration, was indicative of having near normal movement performance and quality when measured with kinematics. These findings are novel and show that assessment of activity capacity, e.g., by ARAT, can be used to identify individuals in which an additional assessment of kinematics might be valuable in order to quantify specific deficits in movement performance and movement quality.

Previous kinematic studies in SCI population have predominantly recruited individuals with complete cervical injury at specific neurological level, commonly C5, C6, and C7 SCI level (15–19). The tasks included in the kinematic studies vary, but often comprise some level of pointing, reaching or grasping (15–19, 35, 36). Kinematic parameters of movement performance during a more complex purposeful daily tasks, such as drinking, have previously only been described in individuals with complete cervical (C6 and C7) SCI (20).

### Movement Time and Smoothness

In the current study, the movement time was significantly different from non-disabled controls in those with limited upper extremity functioning. This finding is in line with previous kinematic studies in individuals with complete cervical SCI (15–17, 20), but extends it further by showing that movement time is prolonged also in a more diverse SCI population. Furthermore, movement time was longer in each separate phase that required transport of the arm and hand in space together with glass manipulation. Longer movement times in reaching phase have been previously reported in C6 and C7 level complete SCI (16, 17, 20), although the prolonged time in the transport phases, as demonstrated in the current study, has not been shown before. The sample sizes in previous studies were small, which might have masked the possible significant differences as seen in the current study.

Movement smoothness, measured as number movement units, was the kinematic measure that showed large effect sizes (0.13–0.17). Ideally, a movement velocity profile has a bell-shape and presents only a single predominant velocity peak somewhere in the middle of a movement phase (26). The total number of movement units, comprising four movement phases will then have a minimum value of four peaks. In the current study, the non-disabled controls and the full functioning group both had in average six movement peaks during the entire drinking task. Individuals with limited upper extremity functioning after SCI demonstrated, however, 2.5 times more movement units (mean 15.2 units) than the controls. This means that the movements in the limited functioning group were more segmented and presented multiple subsequent accelerations and decelerations. Increased number of movement units was also reported in individuals with complete cervical SCI (C5–C8) both in reach-to-grasp (15) and drinking task (21), but no differences were found between the C6 and C7 groups (21). Hand path curvature ratio, another measure of smoothness, was also increased during a reach-to-touch task when performed without trunk support in a small sample of five individuals with complete SCI with varying levels of SCI (C7–T4) (36). The results of the current study extend the previous research showing that movement smoothness is a sensitive measure for quantification of quality of movement in persons with limited upper extremity functioning after SCI.

### Movement Velocity and Strategy

Independent from functioning level, three kinematic measures related to movement velocity in reaching did not differ from the non-disabled group. This finding was somewhat surprising, particularly for the group with limited functioning. Then again, analogous results for the peak velocity and time to peak velocity have been reported for individuals with complete C5–C7 SCI (15–17, 20, 37).

In contrast to other clinical populations, the velocity-related kinematics have shown to be decreased in people with stroke (23, 38). This finding provides an interesting insight into understanding the differences in motor control and movement execution between stroke and SCI. While people with stroke exhibit slow movements together with lower peak tangential and angle velocity (23, 38), in SCI only movement time seems to be affected. This discrepancy can partly be related to the nature of the injury, suggesting that the injury in the brain rather than in the spinal cord might influence the velocity related kinematics. The ability to scale velocity production in reaching indicates that people with SCI have adequate movement planning and movement initiation. However, due to the deficits in grasping and object manipulation the movement time is still prolonged. In a previous study with five individuals with C6 complete SCI, the reaching trajectories in a grasping task were higher than in controls even when there were no differences in peak velocities (17). The authors concluded that the altered movement pattern could be attributed to the acquisition of new motor strategies to compensate for grasping impairment rather than to compensate the triceps paralysis (17). The same reasoning might be extended to the near normal angular velocity of the elbow extension as seen in the current study, although, here, we have not found any previous data in SCI populations. Future studies are, however, needed for improved understanding of these discrepancies between clinical populations.

### Movement Pattern

Individuals with limited upper extremity functioning used a larger wrist angle (dorsal flexion) than non-disabled controls while grasping and transporting the glass. The arm was more abducted during drinking, and the trunk displacement was larger compared with controls. Differences in wrist angle have been shown previously in individuals with complete C6 or C7 SCI (15, 16, 20) and can partly be accounted for the compensatory grasping strategy when the passive “tenodesis” is used for grasping.

Abduction of the shoulder is another compensatory strategy used when bringing the drinking cup to the mouth. In contrast to our results, differences from non-disabled controls were not detected in a small sample with complete C6 or C7 SCI (15, 20). In another study including five individuals with C7–T4 SCI, an increased trunk displacement was, however, evident when reaching within 80% of the arm's reach (36). Interestingly, the only kinematic variable of the full functioning group that was significantly different (moderate ES of 0.06) from controls was the arm abduction angle used during the drinking phase. It is difficult to know the reason for that finding, but possibly, it could have been connected to the trunk stabilization strategy.

In the current study, the compensatory trunk displacement was twice as large in individuals with limited upper extremity functioning after SCI when compared with controls. In people with stroke, the increased forward movement of the trunk is commonly observed concurrently with decreased elbow extension during reaching (23). However, in the current cohort with SCI, the elbow extension was not different from the non-disabled controls, which indicates that the redundant movement of the trunk could be part of a stabilizing movement strategy.

The inter-joint coordination, i.e., correlation between shoulder flexion and elbow extension in reaching phase, was significantly different from non-disabled controls (moderate ES = 0.06). In contrast to our study, no significant differences from non-disabled controls were observed for inter-joint coordination of drinking task in persons with complete C6 or C7 level of SCI (20). Possibly it could be that the small sample size with eight participants in each group could have masked the possible statistical inferences in this abovementioned study.

### Strengths and Limitations

The sample size of this study was large compared with previous kinematic studies in SCI populations (sample sizes in previous studies vary from 4 to 20 participants). We aimed to include everyone able to perform the drinking task, regardless whether the cervical or thoracic SCI was complete or incomplete or whether the participants had undergone hand surgery or not. This resulted in a neurologically diverse but representative sample of people with SCI, which strengthens the external validity of the study. Consequently, the upper extremity functioning varied between the participants with SCI, but it also varied between the arms in the same person. The functioning level of each upper extremity, instead of neurological injury level, was therefore used to form two separate groups: one group with near normal level functioning and the other with limited functioning. As expected, the proportion of cervical complete SCI was larger in the group with limited functioning, but even in the full functioning group, the injury level and completeness varied. This needs to be considered while generalizing the results from the current study. It means that, independent of the SCI level or completeness, the deficits shown in kinematics will be valid for those with limited functioning at activity capacity level. In the current study the limited function was defined as having less than a full score on ARAT. In future studies it would be valuable to investigate further the impact of different grades of functioning on kinematic measures to determine which specific impairments or limitations in activity performance are correlated to specific movement deficits measured with kinematics.

A natural common everyday task, drinking from a glass, was selected for kinematic analysis, which also strengthens the ecological validity of the study. In addition, the kinematic measures were kept simple and straightforward to facilitate the clinical interpretation of the results. However, future studies are needed to evaluate the potential of the kinematic analysis in the clinical decision making.

A limitation of this study is that individuals who could not perform the unimanual drinking task at least with one arm were not included. A different drinking cup (vine glass, or coffee mug type) was used in four participants with SCI that allowed them to execute the drinking task despite difficulties in grasping. This adaptation might have influenced the results, but was considered to be minimal due to low number of cases. Another limitation of the study is that the single markers placed on anatomical landmarks, as used in the current study, will not allow calculation of joint rotations e.g., in the wrist or elbow joints. A more complex biomechanical model with multiple markers on each body segment would allow a more detailed analysis of joint angles, but it will significantly increase the time required for experimental set-up and analysis. Our findings show that clinically relevant data for SCI population can be extracted using a single marker setup. It is also worth to notice that most of the kinematics analyzed in this study were derived from the reaching phase. The reaching phase is commonly analyzed in kinematic studies using different tasks and will in this way allow comparison with other studies. It is well-established that kinematics are dependent on the goal of the task, and therefore, it is important that the person has to perform a full purposeful task of drinking and not only the reaching for the glass.

## Conclusion

This study demonstrated that movement time, smoothness, and movements of trunk and arm were altered compared with non-disabled controls in people with limited upper extremity functioning after a cervical or thoracic, complete, or incomplete SCI. The peak velocity and acceleration time in reaching were, however, near normal. The study provides reliable and robust results applicable to a representative population of individuals with established SCI.

Taken the recent trend toward increased proportion of incomplete SCI resulting in a more diverse patient population, more studies with larger variety of injuries are needed. Today, the kinematic studies of upper extremity tasks have, to a large part only, investigated people with complete specific cervical level of injury. Furthermore, the importance to consider the functioning level, and not only the neurological level, or completeness of injury becomes even more relevant. The clinical functional assessments, like ARAT, should be used to get a better understanding of upper extremity functioning. The results suggest that kinematic analysis might be useful for those with limited functioning in order to get a better understanding of the specific movement impairments in daily tasks after SCI.

## Data Availability Statement

The raw data supporting the conclusions of this article will be made available by the authors, upon reasonable request.

## Ethics Statement

The studies involving human participants were reviewed and approved by the Swedish Ethical Review Authority (408-17). The patients/participants provided their written informed consent to participate in this study.

## Author Contributions

LL conducted the data collection, performed the data analysis, and drafted the manuscript. MA contributed to the data collection, analysis, and drafting. MA, TR, and KS revised the manuscript critically for intellectual content. All authors made substantial contributions to the concept, design, interpretation of the data, read, and approved the final manuscript.

## Funding

This study was funded by the Swedish Research Council (VR 2017-00946), the Swedish Society for Medical Research (S19-0074), the Swedish state under an agreement between the Swedish government and the county councils, the ALF agreement (ALFGBG-718711, ALFGBG-879111, and ALFGBG-826331), and Promobilia, the local Research and Development Board for Gothenburg and Södra Bohuslän, Sahlgrenska, University Hospital Research Foundation.

## Conflict of Interest

The authors declare that the research was conducted in the absence of any commercial or financial relationships that could be construed as a potential conflict of interest.

## Publisher's Note

All claims expressed in this article are solely those of the authors and do not necessarily represent those of their affiliated organizations, or those of the publisher, the editors and the reviewers. Any product that may be evaluated in this article, or claim that may be made by its manufacturer, is not guaranteed or endorsed by the publisher.

## References

[1] Nijendijk JH Post MW van Asbeck FW. Epidemiology of traumatic spinal cord injuries in the Netherlands in 2010. Spinal Cord. (2014) 52:258–63. 10.1038/sc.2013.180 24445971

[2] Hagen EM Eide GE Rekand T Gilhus NE Gronning M. A 50-year follow-up of the incidence of traumatic spinal cord injuries in Western Norway. Spinal Cord. (2010) 48:313–8. 10.1038/sc.2009.133 19823192

[3] Calabro FJ Perez MA. Bilateral reach-to-grasp movement asymmetries after human spinal cord injury. J Neurophysiol. (2016) 115:157–67. 10.1152/jn.00692.2015 26467518 PMC4760481

[4] Devivo MJ. Epidemiology of traumatic spinal cord injury: trends and future implications. Spinal Cord. (2012) 50:365–72. 10.1038/sc.2011.178 22270188

[5] World Health Organization. International Classification of Functioning, Disability and Health: ICF. Geneva: World Health Organization (2001). p. 299.

[6] Kirshblum SC Burns SP Biering-Sorensen F Donovan W Graves DE Jha A. International standards for neurological classification of spinal cord injury (revised 2011). J Spinal Cord Med. (2011) 34:535–46. 10.1179/204577211X13207446293695 22330108 PMC3232636

[7] Steeves JD Lammertse DP Kramer JL Kleitman N Kalsi-Ryan S Jones L. Outcome measures for acute/subacute cervical sensorimotor complete (AIS-A) spinal cord injury during a phase 2 clinical trial. Top Spinal Cord Inj Rehabil. (2012) 18:1–14. 10.1310/sci1801-1 23239927 PMC3519288

[8] Post MW Kirchberger I Scheuringer M Wollaars MM Geyh S. Outcome parameters in spinal cord injury research: a systematic review using the International Classification of Functioning, Disability and Health (ICF) as a reference. Spinal Cord. (2010) 48:522–8. 10.1038/sc.2009.177 20048752

[9] Burridge J Alt Murphy M Buurke J Feys P Keller T Klamroth-Marganska V. A systematic review of international clinical guidelines for rehabilitation of people with neurological conditions: what recommendations are made for upper limb assessment? Front Neurol. (2019) 10:567. 10.3389/fneur.2019.00567 31293493 PMC6603199

[10] Chan CWL Miller WC Querée M Noonan VK Wolfe DL. The development of an outcome measures toolkit for spinal cord injury rehabilitation. Canad J Occup Therapy. (2017) 84:119–29. 10.1177/0008417417690170 28378605

[11] Thorsen R Binda L Chiaramonte S Dalla Costa D Redaelli T Occhi E. Correlation among lesion level, muscle strength and hand function in cervical spinal cord injury. Eur J Phys Rehabil Med. (2014) 50:31–8. 23820875

[12] Harvey LA Dunlop SA Churilov L Galea MP Spinal Cord Injury Physical Activity Hands On Trial C. Early intensive hand rehabilitation is not more effective than usual care plus one-to-one hand therapy in people with sub-acute spinal cord injury ('Hands On'): a randomised trial. J Physiother. (2017) 63:197–204. 10.1016/j.jphys.2017.08.005 28970100

[13] Schwarz A Kanzler CM Lambercy O Luft AR Veerbeek JM. Systematic review on kinematic assessments of upper limb movements after stroke. Stroke. (2019) 50:718–27. 10.1161/STROKEAHA.118.023531 30776997

[14] Kwakkel G van Wegen EEH Burridge JH Winstein CJ van Dokkum LEH Alt Murphy M. Standardized measurement of quality of upper limb movement after stroke: consensus-based core recommendations from the second stroke recovery and rehabilitation roundtable. Neurorehabil Neural Repair. (2019) 33:951–8. 10.1177/1545968319886477 31660781

[15] Cacho EW de Oliveira R Ortolan RL Varoto R Cliquet A Jr. Upper limb assessment in tetraplegia: clinical, functional and kinematic correlations. Int J Rehabil Res. (2011) 34:65–72. 10.1097/MRR.0b013e32833d6cf3 20805758

[16] Mateo S Revol P Fourtassi M Rossetti Y Collet C Rode G. Kinematic characteristics of tenodesis grasp in C6 quadriplegia. Spinal Cord. (2013) 51:144–9. 10.1038/sc.2012.101 22945744

[17] Laffont I Briand E Dizien O Combeaud M Bussel B Revol M. Kinematics of prehension and pointing movements in C6 quadriplegic patients. Spinal Cord. (2000) 38:354–62. 10.1038/sj.sc.3100999 10889564

[18] Hoffmann G Laffont I Hanneton S Roby-Brami A. How to extend the elbow with a weak or paralyzed triceps: control of arm kinematics for aiming in C6-C7 quadriplegic patients. Neuroscience. (2006) 139:749–65. 10.1016/j.neuroscience.2005.12.018 16448777

[19] Jacquier-Bret J Rezzoug N Vallier JM Tournebise H Gorce P. Reach to grasp kinematics and EMG analysis of C6 quadriplegic subjects. Conf Proc IEEE Eng Med Biol Soc. (2009) 2009:5934–7. 10.1109/IEMBS.2009.5334747 19965061

[20] de los Reyes-Guzman A Gil-Agudo A Penasco-Martin B Solis-Mozos M del Ama-Espinosa A Perez-Rizo E. Kinematic analysis of the daily activity of drinking from a glass in a population with cervical spinal cord injury. J Neuroeng Rehabil. (2010) 7:41. 10.1186/1743-0003-7-41 20727139 PMC2936358

[21] de los Reyes-Guzman A Dimbwadyo-Terrer I Perez-Nombela S Monasterio-Huelin F Torricelli D Pons JL. Novel kinematic indices for quantifying movement agility and smoothness after cervical Spinal Cord Injury. NeuroRehabilitation. (2016) 38:199–209. 10.3233/NRE-161311 26923358

[22] von Elm E Altman DG Egger M Pocock SJ Gotzsche PC Vandenbroucke JP. The Strengthening the Reporting of Observational Studies in Epidemiology (STROBE) statement: guidelines for reporting observational studies. J Clin Epidemiol. (2008) 61:344–9. 10.1016/j.jclinepi.2007.11.008 18313558

[23] Alt Murphy M Willen C Sunnerhagen KS. Kinematic variables quantifying upper-extremity performance after stroke during reaching and drinking from a glass. Neurorehabil Neural Repair. (2011) 25:71–80. 10.1177/1545968310370748 20829411

[24] Alt Murphy M Murphy S Persson HC Bergstrom UB Sunnerhagen KS. Kinematic analysis using 3d motion capture of drinking task in people with and without upper-extremity impairments. J Vis Exp. (2018) 57228. 10.3791/57228 29658937 PMC5933268

[25] Frykberg GE Grip H Alt Murphy M. How many trials are needed in kinematic analysis of reach-to-grasp?—a study of the drinking task in persons with stroke and non-disabled controls. J NeuroEng Rehabil. (2021) 18:101. 10.1186/s12984-021-00895-3 34130716 PMC8207615

[26] Kamper DG McKenna-Cole AN Kahn LE Reinkensmeyer DJ. Alterations in reaching after stroke and their relation to movement direction and impairment severity. Arch Phys Med Rehabil. (2002) 83:702–7. 10.1053/apmr.2002.32446 11994811

[27] Lyle RC. A performance test for assessment of upper limb function in physical rehabilitation treatment and research. Int J Rehabil Res. (1981) 4:483–92. 10.1097/00004356-198112000-00001 7333761

[28] Yozbatiran N Der-Yeghiaian L Cramer SC. A standardized approach to performing the action research arm test. Neurorehabil Neural Repair. (2008) 22:78–90. 10.1177/1545968307305353 17704352

[29] Hsieh CL Hsueh IP Chiang FM Lin PH. Inter-rater reliability and validity of the action research arm test in stroke patients. Age Ageing. (1998) 27:107–13. 10.1093/ageing/27.2.107 16296669

[30] Nordin A Alt Murphy M Danielsson A. Intra-rater and inter-rater reliability at the item level of the Action Research Arm Test for patients with stroke. J Rehabil Med. (2014) 46:738–45. 10.2340/16501977-1831 24953235

[31] Kowalczewski J Chong SL Galea M Prochazka A. In-home tele-rehabilitation improves tetraplegic hand function. Neurorehabil Neural Repair. (2011) 25:412–22. 10.1177/1545968310394869 21372246

[32] Biering-Sørensen F Bryden A Curt A Friden J Harvey LA Mulcahey MJ. International spinal cord injury upper extremity basic data set. Spinal Cord. (2014) 52:652–7. 10.1038/sc.2014.87 24891012

[33] Catz A Itzkovich M Tesio L Biering-Sorensen F Weeks C Laramee MT. A multicenter international study on the Spinal Cord Independence Measure, version III: Rasch psychometric validation. Spinal Cord. (2007) 45:275–91. 10.1038/sj.sc.3101960 16909143

[34] Pallant J. SPSS Survival Manual: A Step by Step Guide to Data Analysis Using IBM SPSS, 5th ed. Maidenhead: McGraw-Hill (2013). p. 354.

[35] Mateo S Di Rienzo F Reilly KT Revol P Delpuech C Daligault S. Improvement of grasping after motor imagery in C6-C7 tetraplegia: a kinematic and MEG pilot study. Restor Neurol Neurosci. (2015) 33:543–55. 10.3233/RNN-140466 26409412

[36] Reft J Hasan Z. Trajectories of target reaching arm movements in individuals with spinal cord injury: effect of external trunk support. Spinal Cord. (2002) 40:186–91. 10.1038/sj.sc.3101277 11965557

[37] Robinson MA Hayes SJ Bennett SJ Barton GJ Elliott D. Sensory-motor equivalence: manual aiming in C6 tetraplegics following musculotendinous transfer surgery at the elbow. Exp Brain Res. (2010) 206:81–91. 10.1007/s00221-010-2400-6 20809244

[38] Alt Murphy M Häger CK. Kinematic analysis of the upper extremity after stroke – how far have we reached and what have we grasped? Phys Ther Rev. (2015) 20:137–55. 10.1179/1743288X15Y.0000000002

