# Supplementary material for: Quantifying an Upper Extremity Everyday Task With 3D Kinematic Analysis in People With Spinal Cord Injury and Non-disabled Controls
Source: Front Neurol. 2021 Oct 15;12:755790. doi: 10.3389/fneur.2021.755790 (PMC8555709; doi:10.3389/fneur.2021.755790)
Supplement: Supplementary file 1 [file Table_1.DOCX]

Supplementary Table

Supplementary Table. Clinical characteristics shown separately for each tested upper extremity for all participants with limited (shaded rows) and full (unshaded rows) upper extremity functioning.

|  |  | ISNCSCI classification | | | | Action Research Arm Test | | | | Other upper extremity clinical characteristics | | | | |
| --- | --- | --- | --- | --- | --- | --- | --- | --- | --- | --- | --- | --- | --- | --- |
| ID | Tested arm | AIS  (A-D) | Sensory  level | Motor  level | Motor score  (0-25) | Grasp  (0-18) | Grip  (0-12) | Pinch  (0-18) | Gross  (0-9) | Complications | Assistive devices | Surgery | Contracture | Spasticity  drugs |
| 1 | ND | D | C6 | C5 | 18 | 18 | 9 | 6 | 7 | Extensive | Never | c | No | Yes |
| 1 | D | D | C4 | C4 | 18 | 16 | 8 | 6 | 3 | Extensive | Never | c | Yes | Yes |
| 2 | ND | C | T4 | T4 | 25 | 18 | 11 | 18 | 9 | Min | Never | No | No | No |
| 2 | D | C | T4 | T4 | 25 | Full functioning | | | | Min | Never | No | No | No |
| 3 | ND | C | C5 | C6 | 20 | Full functioning | | | | Min | Never | No | No | No |
| 3 | D | C | C5 | C6 | 14 | 16 | 11 | 11 | 8 | Mod | Never | a,b,c | Yes | No |
| 4 | ND | D | C5 | C5 | 17 | 17 | 10 | 16 | 9 | Min | Never | No | No | No |
| 4 | D | D | C7 | C7 | 25 | Full functioning | | | | Min | Never | No | No | No |
| 5 | ND | E | No level | No level | 25 | Full functioning | | | | Min | Never | No | No | No |
| 5 | D | E | No level | No level | 25 | Full functioning | | | | Min | Never | No | No | No |
| 6 | ND | A | T4 | T4 | 25 | Full functioning | | | | Min | Never | No | No | No |
| 6 | D | A | T4 | T4 | 25 | 18 | 12 | 18 | 7 | Min | Never | No | No | No |
| 7 | ND | A | C3 | C3 | 5 | 6 | 4 | 2 | 5 | Mod | Daily | No | No | Yes |
| 7 | D | A | C3 | C3 | 5 | 8 | 4 | 2 | 5 | Min | Daily | No | Yes | Yes |
| 8 | ND | A | T2 | T2 | 25 | 16 | 9 | 18 | 7 | Min | Daily | No | No | Yes |
| 8 | D | A | T2 | T2 | 25 | 18 | 9 | 18 | 9 | Min | Daily | No | No | Yes |
| 9 | ND | B | C7 | C6 | 15 | 17 | 8 | 6 | 7 | Min | Never | No | Yes | No |
| 9 | D | B | C8 | C6 | 18 | 17 | 8 | 16 | 9 | Min | Never | d | Yes | No |
| 10 | ND | A | C6 | C6 | 14 | 8 | 6 | 4 | 9 | Min | Daily | No | No | No |
| 10 | D | A | C6 | C6 | 15 | 12 | 6 | 4 | 9 | Min | Daily | No | No | No |
| 11 | ND | A | C4 | C4 | 11 | 8 | 6 | 2 | 5 | Min | Weekly | a,b | No | No |
| 11 | D | A | C4 | C4 | 11 | 6 | 4 | 0 | 7 | Min | Weekly | a,b | No | No |
| 12 | ND | A | T8 | T8 | 25 | Full functioning | | | | Min | Never | No | No | No |
| 12 | D | A | T7 | T7 | 25 | Full functioning | | | | Min | Never | No | No | No |
| 13 | ND | A | C8 | T1 | 24 | Full functioning | | | | Min | Never | No | No | No |
| 13 | D | A | C8 | T1 | 24 | Full functioning | | | | Min | Never | No | No | No |
| 14 | ND | B | T3 | T3 | 25 | 18 | 11 | 18 | 9 | Min | Never | No | No | No |
| 14 | D | B | T3 | T3 | 25 | Full functioning | | | | Min | Never | Other | No | No |
| 15 | ND | A | C4 | C4 | 25 | 18 | 12 | 18 | 7 | Mod | Never | d | No | Yes |
| 15 | D | A | C5 | C8 | 24 | 18 | 12 | 18 | 6 | Mod | Never | No | No | Yes |
| 16 | ND | B | C4 | C4 | 17 | 18 | 12 | 18 | 7 | Mod | Never | No | No | Yes |
| 16 | D | B | C4 | C4 | 25 | Full functioning | | | | Min | Never | No | No | Yes |
| 17 | ND | E | No level | No level | 25 | Full functioning | | | | Min | Never | No | No | No |
| 17 | D | E | No level | No level | 25 | Full functioning | | | | Min | Never | No | No | No |
| 18 | ND | A | C6 | C7 | 14 | 13 | 8 | 4 | 8 | Mod | Weekly | b, c, d,e | No | No |
| 18 | D | A | C5 | C6 | 11 | 6 | 8 | 2 | 8 | Extensive | Weekly | No | No | No |
| 19 | ND | D | C4 | C4 | 20 | 12 | 8 | 12 | 5 | Mod | Never | No | No | No |
| 19 | D | D | C4 | C4 | 25 | Full functioning | | | | Min | Never | No | No | No |
| 20 | ND | B | T2 | T2 | 25 | Full functioning | | | | Min | Never | No | No | No |
| 20 | D | B | T2 | T2 | 25 | Full functioning | | | | Min | Never | No | No | No |
| 21 | ND | C | L3 | L2 | 25 | Full functioning | | | | Min | Never | No | No | No |
| 21 | D | C | T12 | T12 | 25 | Full functioning | | | | Min | Never | No | No | No |
| 22 | ND | D | C4 | C4 | 25 | Full functioning | | | | Min | Never | Other | No | No |
| 22 | D | D | C4 | C4 | 25 | Full functioning | | | | Min | Never | No | No | No |
| 23 | D* | B | C3 | C3 | 9 | 4 | 4 | 0 | 8 | Mod | Never | a,b,c | No | No |
| 24 | ND | D | C2 | C2 | 23 | 17 | 11 | 17 | 9 | Mod | Never | No | No | No |
| 24 | D | D | C3 | C3 | 20 | 17 | 10 | 16 | 7 | Mod | Never | c | No | No |
| 25 | ND | D | C2 | C2 | 21 | Full functioning | | | | Min | Never | No | No | No |
| 25 | D | D | C2 | C2 | 21 | 18 | 12 | 17 | 7 | Mod | Never | No | No | No |
| 26 | ND | D | C4 | C4 | 20 | 15 | 9 | 15 | 7 | Min | Never | No | No | No |
| 26 | D | D | C4 | C4 | 25 | Full functioning | | | | Min | Never | No | No | No |
| 27 | ND | D | T10 | T10 | 25 | Full functioning | | | | Min | Never | No | No | No |
| 27 | D | D | T10 | T10 | 25 | Full functioning | | | | Min | Never | Other | No | No |
| 28 | ND | C | T11 | T11 | 25 | Full functioning | | | | Min | Never | No | No | No |
| 28 | D | C | T11 | T11 | 25 | Full functioning | | | | Min | Never | No | No | No |
| 29 | ND | A | C4 | C4 | 18 | 17 | 8 | 0 | 9 | Min | Never | a,e | Yes | No |
| 29 | D | A | C4 | C4 | 18 | 17 | 8 | 4 | 9 | Mod | Never | b,e | No | No |

Abbreviations: ISNCSCI, International Standards for Neurological Classification of Spinal Cord Injury; ISCoS, International Spinal Cord Society, AIS, American Spinal Injury Association (ASIA) Impairment scale; Motor score, tested upper extremity (0–25); a, transfer in elbow or wrist; b, restoration pinch and/or grasp; c, release; d, other soft tissue reconstruction; e, osteotomy with arthrodesis; D, dominant arm; ND, non-dominant arm; * only dominant arm was tested in this individual; In 9 participants the upper extremity functioning was categorized as limited in one arm and full in the other arm according to the Action Research Arm Test.
